# Rehydrating efficacy of maple water after exercise-induced dehydration

**DOI:** 10.1186/s12970-019-0273-z

**Published:** 2019-02-11

**Authors:** Alexs Matias, Monique Dudar, Josip Kauzlaric, Kimberly A. Frederick, Shannon Fitzpatrick, Stephen J. Ives

**Affiliations:** 1 Health and Human Physiological Sciences Department, Skidmore College, 815 N. Broadway, Saratoga Springs, NY 12866 USA; 2 Physics Department, Saratoga Springs, USA; 3 Chemistry Department, Skidmore College, Saratoga Springs, NY USA; 4 Exercise Science Department, George Washington University, Washington, USA

**Keywords:** Maple sap, Hydration, Thirst, Antioxidants, Electrolytes

## Abstract

**Abstract:**

Dehydration impairs physiological function and physical performance, thus understanding effective rehydration strategies is paramount. Despite growing interest in natural rehydrating beverages, no study has examined maple water (MW).

**Purpose:**

To investigate the rehydrating efficacy of MW after exercise-induced dehydration.

**Methods:**

Using a single-blind, counterbalanced, crossover design, we compared the rehydrating efficacy of MW vs. maple-flavored bottled water (control) in 26 young healthy (22 ± 4 yrs., 24 ± 4 kg/m^2^) males (*n* = 13) and females (*n* = 13) after exercise-induced dehydration (~ 2.0%ΔBody Weight [BW]) in the heat (30 °C, 50% relative humidity [RH]). Hydration indicators (BW, salivary and urine osmolality [SOsm/UOsm], urine specific gravity [USG], urine volume [UV], urine color [UC]), thirst, fatigue, and recovery (heart rate [HR)], and HR variability [HRV]) were taken at baseline, post-exercise, 0.5, 1, and 2 h post-consumption of 1 L of MW or control.

**Results:**

Following similar dehydration (~ 2%ΔBW), MW had no differential (*p* > 0.05) impact on any measure of rehydration. Likely due to greater beverage osmolality (81 ± 1.4 vs. 11 ± 0.7 mOsmol/kg), thirst sensation remained 12% higher with MW (*p* <  0.05). When sex was considered, females had lower UV, elevated UOsm (*p* < 0.05), trends for higher ΔBW, USG, but similar SOsm. Analysis of beverages and urine for antioxidant potential (AP) revealed a four-fold greater AP in MW, which increased peak urine AP (9.4 ± 0.7 vs. 7.6 ± 1.0 mmol, MW vs. control, *p* <  0.05).

**Conclusion:**

Electrolyte-containing MW, was similar in effectiveness to water, but has antioxidant properties. Furthermore, trends for sex differences were discovered in urinary, but not salivary, hydration markers, with discrepancies in kinetics between fluid compartments both warranting further study.

## Introduction

Exhaustive physical work, common in athletics and certain occupations (i.e. firefighting), imposes considerable physiological demands, which is exacerbated by impaired heat dissipation due to environmental heat stress [1–4]. To mitigate excessive elevations in body temperature, heat is dissipated via multiple mechanisms, such as perspiration, which is augmented in the heat [3–6]. Specifically, during moderate intensity exercise in the heat, sweat-induced water loss can be profound, as water losses of 0.8–2.0 L/h are not uncommon, in addition to loss of water conserving mineral salts (i.e. Na^+^, K^+^, Ca^++^, etc.) that act as electrolytes [4, 6, 7]. Dehydration is an imbalance between fluid ingestion and loss, which may lead to dysfunction [4], illness [8], and likely increases susceptibility to heat related illness or injury [3, 9]. Further, excessive electrolyte loss, or excessive water intake, can lead to hyponatremia [10], as many physiological processes are dependent on a stable hydromineral balance. Dehydration-induced BW losses of > 1–2% have been documented to impair cognitive function and mood, likely increasing fatigue [11–13], which consequently might also, at least in part, impair aerobic exercise performance [4, 14, 15]. Thus, excessive sweat-induced hydromineral loss is a serious concern for those engaging in intensive physical activity, prompting investigation on effective rehydration strategies.

Indeed, in an attempt to combat loss of and/or replenish hydromineral stores, there is a relatively long history of athletes seeking the ergogenic aid of carbohydrate-electrolyte rich sport beverages, which have been demonstrated to promote rehydration with varying degrees of success [16–21]. However, issues such as, caloric content (e.g. ~ 150–300 Kcal/bottle) in those cognizant about weight and body composition [22], the use of food dyes, artificial additives and sweeteners, and possible gastrointestinal (GI) discomfort have raised concerns regarding the use of carbohydrate-electrolyte rich beverages [23–25]. Thus, anecdotally, there is a growing interest for natural alternatives to artificial carbohydrate-electrolyte rich beverages.

In this regard, coconut water (CW) has recently gained popularity due to its naturally occurring mineral electrolytes (i.e. K^+^, Na^+^, Cl^−^), antioxidants, and carbohydrates [26–29]. However, Kalman et al. [29], documented no significant differences between CW and a carbohydrate-electrolyte rich beverage on measures of fluid retention, plasma osmolality, urine specific gravity (USG), and time to exhaustion following 2% BW loss. Similarly, following 3% BW loss, no differences in rehydration were observed between a sports drink, CW, and sodium-enriched CW, although all three beverages were superior to plain water [27]. Concerns over the sustainability of CW, which is sourced from tropical regions, may also impede its widespread use.

Anecdotally, maple water (MW; sap from maple trees) has been suggested as an effective natural alternative for rehydration [30] and consumption of MW has increased dramatically and is expected to continue such growth. MW is an organically sourced beverage that contains half the calories of CW, as well as naturally occurring polyphenolic compounds, antioxidants, prebiotics, electrolytes, and malate, a Krebs cycle intermediate with proposed physiological benefit [31, 32]; enhanced aerobic energy production, corresponding reduction in muscle fatigue and augmented endurance. MW is purported to have antioxidant effects either directly through endogenous compounds or indirectly via manganese (ea. serving of MW has 40% RDI) which acts as a co-factor in superoxide dismutase (SOD), specifically for the SOD2 subtype, and/or may improve vitamin absorption. While the composition and potential health impacts of maple syrup have been explored [33, 34], to the best of our knowledge, no studies have investigated the characteristics of MW, nor the potential rehydrating effects, despite growing interest.

Further, given prior work which demonstrated similar physiological response to exercise under eu- and dehydrated states between the sexes [35] coupled with evidence suggesting that menstrual cycle phase has no impact on rehydration after exercise-induced dehydration [21], we sought to include females. Though, Eijsvogels et al. demonstrated that sex differences might exist in dehydration, which appear to be in part to differences in fluid intake between men and women and can result in altered electrolyte profiles [36], perhaps mediated through sex specific hormone effects on the renin angiotensin aldosterone system [37, 38], resulting in women more likely to maintain plasma osmolality, and men more likely to become hypernatremic [36]. Though, studies of rehydration responses in males and females under matched relative exercise intensity, body weight loss, and controlled fluid intake, are needed.

Accordingly, the primary purpose of the present study was to investigate the efficacy of MW as a rehydration beverage following moderate-intensity cycling performed in a hyperthermic environment. A second aim was to characterize the electrolyte profile, osmolality, antioxidant capacity of MW, and the potential biological impact of MW antioxidant properties. It was hypothesized that ingestion of 1 L of MW following 2% BW loss would enhance physiological and perceptual markers of hydration, reduce fatigue, and enhance recovery over that of maple-flavored bottled water. Finally, we hypothesized there would be no differences in objective or subjective measures of rehydration in response to maple water between men and women.

## Methods

### Subjects and general procedures

Twenty-six, healthy, physically active college-aged (22 ± 1 yrs.) males (*n* = 13) and females (*n* = 13) were recruited by public advertisement and word of mouth from Skidmore College and the greater Saratoga Springs region in New York. Given previous work demonstrating similar physiological response to hypohydration between the sexes [35] coupled with evidence finding no impact of the menstrual cycle phase on rehydration after exercise-induced dehydration [21], we did not exclude females, nor did we control for menstrual cycle phase or oral contraceptive use. To be considered healthy, participants could not have been current or recent (> 6 months not smoking) smokers, and those with any history of cardiovascular, renal, musculoskeletal, or metabolic diseases were excluded. Health history was collected using questionnaires (American College of Sports Medicine Pre-Participation Screening and Physical Activity Readiness Questionnaire [PAR-Q]) to assess for eligibility. Physical activity was defined, in accordance with ACSM, as regularly engaging in a minimum of 30 min of moderate intensity aerobic activity at least 3 days a week [39]. All participants provided written informed consent prior to participation. Approval for this study was granted by the Human Subjects Institutional Review Board (IRB#1608–531) of Skidmore College and is in accordance with the most recent revisions of the Declaration of Helsinki.

To characterize participants, height and weight were measured using standard techniques, and body fat percentage, fat mass, and fat free mass were measured using air displacement plethysmography (BodPod, CosMed, Chicago, IL). Participants were asked to refrain from consuming alcohol or caffeine, or from participating in any strenuous exercise, at least 12 and 24 h (h) prior to testing, respectively. To avoid interference with salivary osmolality measures, participants were not permitted to eat or perform oral hygiene at least 90 min prior to testing, but could consume only water ad libitum up until 15 min prior to arriving to lab. Participants were asked to report to lab well hydrated, and were suggested to consume at least 30 ml/kg/day of water 24 h prior to testing to achieve adequate hydration, which was confirmed upon arrival (USG <  1.020). Those with USG values of > 1.020 were given 500 mL of bottled water to be consumed over 15 min until sufficiently hydrated (USG <  1.020), prior to the commencement of testing. However, this only occurred in 4 of 52 trials total. To avoid the potential confound of food ingestion on fluid retention food intake was prohibited during the rehydration period.

### Study design

**Fig. 1:**
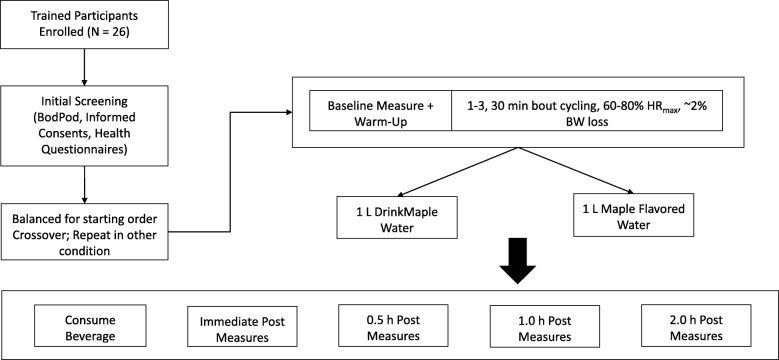
Experimental Design. Using a counterbalanced design, subjects were randomly assigned to either the control (maple flavored water) or maple water (DrinkMaple, MW) for their first visit. Baseline measures of hydration (nude body weight, analog and digital USG, saliva and urine osmolality, urine volume, urine color), perceptual measures of thirst and fatigue, HR and HRV. Following cycling to ~ 2% Dehydration, the same battery of measures were taken immediately post-exercise, 0.5, 1.0, and 2.0 h post consumption of assigned beverage. Participants returned no less than 48 h later to complete the trial in the other condition

The current study was conducted using a counterbalanced, single-blind, placebo-controlled, within subjects crossover design (Fig. 1). Subjects were randomly assigned to consume 1 L of maple flavored water (MFW; control, 0.267 ml of Maple Extract [McCormick & Co Inc., Hunt Valley, MD] per 500 ml bottled water) or Maple Water (MW; DRINKmaple LLC, Concord, MA). Maple extract was used to mimic the flavor and odor of maple water, but provided no nutritional value. To allow for adequate recovery, testing was divided into two days separated by a minimum washout period of at least 48 h, but were typically completed within a week (average time between visits = 166 h). The study protocol was identical for both interventions, with the exception of the beverage consumed. After obtaining immediate post-exercise measures, beverages were consumed, in four, 250 ml dosages, timed 4–7.5 min apart, and served at room temperature to maximize gastric emptying and fluid uptake [40, 41], while minimizing diuresis. Thus, using this metered intake to control for intake pacing, ingestion was completed in no less than 15 min but no more than 30 min. From a practical perspective, an absolute volume of fluid was chosen to assess the rehydrating efficacy of MW, to better represent realistic post-exercise situations where athletes are likely to rehydrate with the bottle of fluid available on hand, rather than the prescribed relative volume intake (e.g. 120–150% BW loss).

### Dehydration protocol

On the first day of testing, following baseline measures, subjects were instructed to cycle (Monark 828E, Health Care Int., WA) at 70% of their age-predicted HR_max_ (HR_max_ = 220 – age in years) in hyperthermic conditions (30 °C and 50% relative humidity [RH]) (Fig. 1). Subjects completed 30 min bouts of cycling until they had lost ~ 2.0% of body weight (BW), and based on previous work in our lab, this threshold was expected to occur in 2 bouts (unpublished observations). To ensure subjects exercised at the designated intensity, heart rate (HR), revolutions per minute (RPM), and resistance (kp) were recorded on a minute-by-minute basis and HR was provided in real time to participants using heart rate monitors (Polar H7) and the Polar Team application (Polar USA, Lake Success, NY) displayed on a screen (iPad, Apple Inc., CA) visible to participants.

### Assessments of hydration

As there is no gold standard measure of hydration [42] and sensitivity varies between method [43] we sought to comprehensively assess the magnitude of dehydration and rehydration. Nude body weight (NBW), urine volume (UV), urine osmolality (UOsm), urine specific gravity (USG), urine color (UC), and salivary osmolality (SOsm) were obtained prior to dehydration, immediately post-dehydration, and at 0.5, 1, and 2 h post-rehydration. Due to institutional limitations measurement of plasma osmolality a critical measure of static hydration, a relatively invasive measure, was not possible, though it may not be sensitive to modest levels of dehydration likely due to tightly regulated homeostatic mechanisms [43, 44]. Additionally, NBW was assessed following every 30-min bout of the dehydration period to establish percent BW lost (Fig. 1).

### Nude body weight (NBW)

Participants were instructed to weigh themselves nude in a private exam room, ensuring to towel off and completely remove any sweat. NBW was used to calculate fluid loss, retention, and to determine number of exercise bouts to be performed. In light of recently published guidelines [45], we performed a reliability analysis of the scale used in our laboratory (model 599KL, Health o Meter), with a reported resolution of 0.1 kg, which revealed high reliability (Cronbachs a = 1.0, Intraclass correlation coefficient = 1.0, *p* <  0.000, *n* = 10 trials, across a range of 8 different weights from 2 to 91 kg, average coefficient of variation [CV] = 0.03%). Given the within subjects design and that HR and workload were matched between trials, non-sweat losses of mass were assumed to be minimal and constant between trials.

### Urine volume

Entire urine voids were collected in 120 ml urine specimen cups and then measured using a graduated cylinder [7, 44]. Urine volume (UV) was also used to calculate cumulative urine output after fluid ingestion (0.5-2H). Following assessment of urine volume, four aliquots were taken from each sample to determine osmolality, specific gravity, color, and antioxidant properties.

### Urine and salivary osmolality (UOsm/SOsm)

UOsm is used to determine the quantity of dissolved particles per unit volume of water in urine and is representative of hydration [7, 44, 46]. SOsm is a measure of the quantity of solutes present in the saliva, and is used to evaluate hydration [46–48]. Using the passive drool technique, participants swallowed to clear their oral cavity, remained seated with their mouth closed, and did not swallow for two minutes while collecting saliva, which was then disposed into a collection vial via a plastic straw. Duplicate measures were taken for at each time point for each participant, via the freezing point depression method using a Fiske 210 Micro Osmometer (Advanced Instruments, Inc., Norwood, MA) and the average calculated. If duplicates were not within 10%, a third measurement was obtained, and the three values were averaged. Coefficient of variations for this measurement in our laboratory was 0.7% in urine and 3.0% in saliva. The instrument was validated with known controls within measurement range prior to and throughout the study.

### Urine specific gravity (USG)

USG compares the density and whole body hydromineral balance of urine to the density of water and is an estimate of hydration [7, 44, 46]. USG was assessed via temperature corrected digital (Reichert TS Meter D, accuracy ±0.0001, Depew, NY) and analog (Fisher Scientific Refractometer, Waltham, MA) refractometry, which were calibrated to manufacturer specifications. The average value was calculated through duplicate measures. If duplicates were not within 10%, a third measurement was obtained, and the three values were averaged. Coefficient of variations for this measurement in our laboratory were 0.0% for digital and 0.1% for analog USG.

### Urine color

For this measurement, an aliquot was taken and compared by a single investigator to a qualitative eight-point urine color scale [7, 49]. All comparisons were conducted within 30 min of obtaining the sample in constant, artificial lighting, with the same background.

### Perceptual measures of thirst and recovery

To understand possible effects of MW on recovery, fatigue, HR, and HR variability (HRV) were recorded prior to dehydration, immediately post-dehydration, and at 0.5, 1, and 2 h post-rehydration (Fig. 1). Assessments of thirst sensation were also recorded at these time points to quantify perceptions of thirst prior to and following beverage consumption.

### Perceptual measures of thirst and fatigue

Thirst sensation was evaluated using a Visual Analog Scale (VAS) prior to dehydration, immediately post-dehydration, and at 0.5, 1, and 2 h post-rehydration. The VAS consisted of a 100 mm long horizontal line with anchor points on either side stating “no thirst” on the far left and “very thirsty” on the far right. Subjects were seated in a resting position and placed a mark at a point on the VAS corresponding to their perception of thirst at the corresponding time point. Thirst sensation was then quantified using the measured distance from the left end of the continuum to the mark made by the subject. The VAS have been documented to be a valid measure of thirst sensation [50]. Fatigue was evaluated using a Visual Analog Scale (VAS). Similar to “Thirst Sensation”, the VAS for fatigue was that anchor points, from left to right, stated “No Fatigue” and “Severe Fatigue”, respectively. The VAS has been demonstrated as a valid and reliable measure of fatigue [51].

### Heart rate variability

After 5 min of rest, In conjunction with HR, measures of HRV (root mean square of the successive difference [RMSSD], natural log transformed RMSSD [LnRMSSD], and standard deviation of the N-N intervals [SDNN]) were obtained with paced breathing rate [52] over 1 min [53] using a HR monitor (Polar HR7, Polar Electro) and while in a seated position. The signal was sent via low energy Bluetooth to a mobile device (iPad, Apple Inc., CA), which was acquired and analyzed using a mobile application (Elite HRV) [54]. In both the clinical and research settings, HRV, a measure of the balance between sympathetic and parasympathetic influence on heart rate [55]. HRV is a more sensitive measurement, than HR alone, to assess recovery [56], and is likely related to hydration status [57].

### Beverage and urine analysis

To better characterize MW and contextualize potential findings, the Maple Water and Control were analyzed for osmolality, electrolyte content, and antioxidant potential. Beverage osmolality was determined in duplicate using the Fiske Osmometer as described above. Content of the following electrolytes: Ca^++^, Na^+^, K^+^, Mg^+^, Cl^−^, phosphate, carbonate, and sulfate, in the beverages were determined using atomic absorbance spectrophotometry (AAnalyst 800, Perkin Elmer, Waltham, MA) for the cations and ion chromatography (Dionex ICS-2100, ThermoFisher) for the anions, with a typical intra-assay CV of 0.1–2%, and linearity of r^2^ > 0.93, in our lab. Antioxidant potential of the Maple Water and Control were analyzed using the Ferric Reducing Ability of Plasma or Antioxidant Power (FRAP) assay developed by Benzie and Strain (5); intra-assay CV = 3.2%, linearity r^2^ = 1.0. To understand the possible effect of the antioxidant capacity of the maple water in vivo, we also measured antioxidant capacity of the urine using the FRAP assay in a subset of participants (*n* = 14), as described above. To determine potential physiological mechanisms [36], namely differences in the renin-angiotensin aldosterone system [37, 38], a subset of male (*n* = 6) and female (n = 6) urine were assayed for renin and aldosterone using standard enzyme linked immunosorbent assay techniques (R&D Systems, Minneapolis, MN) and both assays displayed good linearity (r^2^ = 0.99–1.0) and reliability (cv = 2.9–3.6%).

### Data analysis

Statistical comparisons were performed with the use of commercially available software (SPSS v. 22.0, IBM Inc., Armonk, NY, USA). Paired t-tests were used to identify potential differences in hydration, perceptual, and cardiovascular measures at baseline prior to testing. Factorial repeated measures of analysis of variance (ANOVA) were used to determine if main effects were found in condition (maple water vs. control), and time (baseline, post, 0.5, 1, and 2 h), and any potential condition by time interactions on any of the measured variables. Exploratory data analysis was performed to determine if trial order, irrespective of condition, had any impact and as females were also included in this study, we also performed analysis using sex as a covariate in the factorial ANOVA described above. Tests of normality were performed, if a significant violation was found, a correction was applied to the corresponding degrees of freedom. Alpha was set, a priori, at 0.05 for all comparisons. All data was presented as mean ± standard deviation (SD).

## Results

### Subject characteristics

An overview of subject characteristics can be found in Table 1. When sex differences were explored, there were significant differences observed for weight, height, percent fat mass, percent fat free mass, and fat free mass (*p* < 0.05), with males generally larger, heavier, and having a lower percent body fat. However, there was no sex difference for age (*p* = 0.948).

**Table 1:** Participant Characteristics

Variable	All (*N* = 26)	Female (*n* = 13)	Male (*n* = 13)	*p*-value
Age (yrs.)	22.0 ± 3.5	22.0 ± 3.9	22.0 ± 3.3	0.95
Weight (kg)	70.8 ± 14.2	61.6 ± 9.3	80.7 ± 11.8	0.00
Height (cm)	170.3 ± 11.8	162.5 ± 4.8	178.7 ± 11.1	0.00
Fat Mass (%)	19.8 ± 8.0	23.3 ± 6.5	16.1 ± 8.0	0.03
Fat Free Mass (%)	80.2 ± 8.0	76.7 ± 6.5	83.9 ± 8.0	0.03
Fat Mass (kg)	14.1 ± 6.7	14.7 ± 5.3	13.4 ± 7.9	0.65
Fat Free Mass (kg)	56.6 ± 12.9	46.8 ± 5.8	67.3 ± 9.3	0.00
BMI (kg m^2^)	24.3 ± 3.6	23.3 ± 3.2	25.3 ± 3.8	0.17

Data presented mean ± SD

### Assessments of hydration

**Fig. 2:**
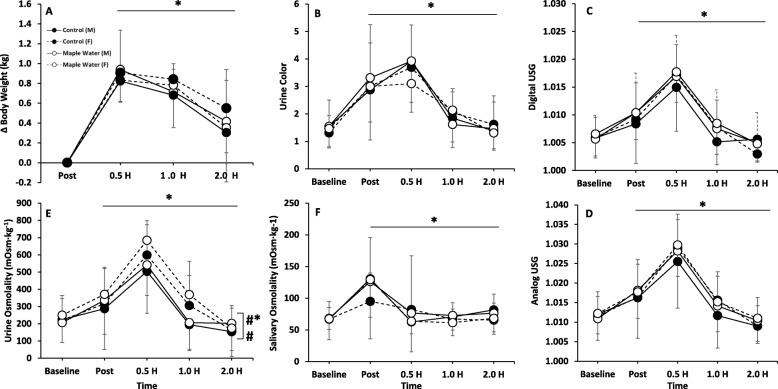
Measurements of Hydration Status at Baseline, 2% Exercise-Induced Dehydration, and after ingestion of Control or Maple Water in Males and Females (*n* = 26). **a** ∆Body weight, **b** Urine Color (1–8 scale), **c** Digital Urine Specific Gravity (USG), **d** Analog USG, **e** Urine Osmolality, **f** Saliva Osmolality. Data are mean ± SD. # *p* < 0.05 main effect for sex, *p < 0.05 main effect for time, #* *p* < 0.05 of interaction sex by time

**Fig. 3:**
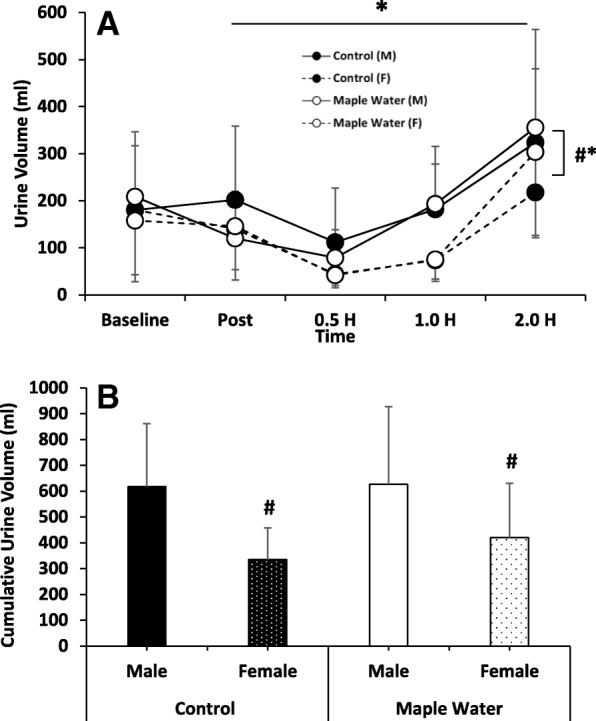
Measurements of Urine Volume (**a**) and Cumulative Urine Volume (0.5–2.0 h, **b**) at Baseline, 2% Exercise-Induced Dehydration, and after ingestion of Control or Maple Water in Males and Females (*n* = 26). Data are mean ± SD. # *p* < 0.05 main effect for sex,*p < 0.05 main effect for time

Baseline assessment of hydration (body weight, UOsm, SOsm, UO, UC, USG) were not different between conditions (Control vs. Maple water, *p* > 0.05). HR and cycling power output were not different between conditions (*p* > 0.05), and thus, actual dehydration achieved was not different between conditions (2 [95% CI: 1.8, 2.2] vs. 2 [95% CI: 1.9, 2.1] %ΔBW, control vs. maple water, *p* > 0.05). We did not observe any impact of trial order, irrespective of condition, on any marker of hydration (*p* > 0.05). There were no significant (*p* > 0.05) condition by time interactions for ΔBW, UOsm, SOsm, UV, UC, Digital USG, or Analog USG (Fig. 2). Also, there was no significant effect of condition (MW vs. control) for ΔBW, UOsm, SOsm, UO, UC, Digital USG, or Analog USG. Cumulative urine output was not different between conditions (476 ± 46 vs. 477 ± 44 ml, *p* > 0.05, Control vs. Maple Water). Though, as expected, significant time effects (*p* < 0.05) were observed for all hydration markers (Fig. 2), where subjects were successfully dehydrated following exercise, and returned to, or near to, baseline by 2.0 h. However, in contrast to the urinary and salivary markers, determination of the ΔBW, suggests that not all the fluid ingested was retained (Fig. 2). Urine volume declined in response to exercise-induced dehydration, rebounded and exceeded baseline in response to fluid ingestion (Fig. 3a, *p* < 0.05). Furthermore, time to peak dehydration differed between urinary and salivary indices; peaks in urinary measures of hydration occurred at 0.5 h, whereas salivary (SOsm) peaked immediately post-exercise (Fig. 2).

When sex was included in the analysis, there was a significant interaction of sex and time (*p* < 0.05, partial η^2^ = 0.1) and a significant main effect of sex observed for UOsm (*p* < 0.05, partial η^2^ = 0.2, Fig. 2). Given similar baseline values for both sexes, females had higher UOsm following exercise, which remained elevated until testing ceased (2.0 h). Although not significant, similar trends were observed for higher USG over time (interaction *p* = 0.09, partial η^2^ = 0.1) and overall (main effect of sex, *p* = 0.08, partial η^2^ = 0.1), though no interaction (*p* > 0.05) or main effect of sex (*p* > 0.05) were observed for UC. Moreover, females had significantly lower urine volumes over time, (interaction *p* < 0.05, partial η^2^ = 0.2) and, on average, tended to have lower urine volumes (Fig. 3a, main effect of sex *p* = 0.09, partial η^2^ = 0.2). Thus, cumulatively, females had lower urine output from time of ingestion to the end of the study period (Fig. 3b, *p* < 0.05). To determine the potential mechanisms for the greater volume retention, a subset of male (*n* = 6) and female (*n* = 6) urine were assayed, and found that females had a tendency for higher urinary renin over time (interaction *p* = 0.10), overall (main effect of sex, *p* = 0.08) and cumulatively (90 ± 26 vs. 56 ± 30 pg/ml, *p* = 0.07). However, no such trends were observed in urinary aldosterone (*p* > 0.05).

### Perceptual measures of thirst and fatigue

**Fig. 4:**
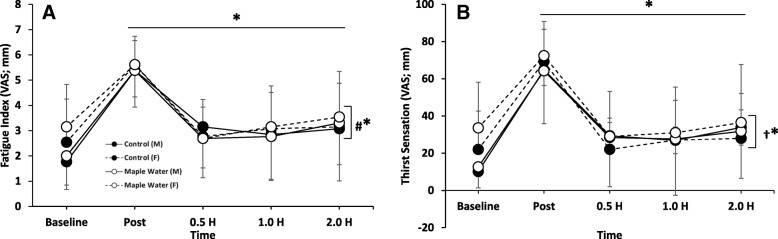
Measurement of Thirst Sensation and Fatigue at baseline, 2% Exercise-Induced Dehydration, and after ingestion of either Control or Maple Water in Males and Females (*n* = 26). Data are mean ± SD. *p < 0.05 main effect for time, † *p* < 0.05 interaction of condition by time, and #* *p* < 0.05 of interaction sex by time

A significant (*p* < 0.05) time effect was observed for perceptions of fatigue and thirst (Fig. 4). Following dehydrating exercise, on average, all participants perceived greater sensations of fatigue and thirst, which returned to, or near to, baseline by 2.0 h. No significant (*p* > 0.05) differences between condition (MW vs. control) were observed. However, a significant interaction of condition and time (*p* < 0.05) was observed for thirst sensation at 0.5, 1.0, and 2.0 h (Fig. 4a), where thirst remained elevated in the MW condition at these time points.

When analyzed according to sex, a significant interaction between sex and time (*p* < 0.05) was observed for fatigue (Fig. 4b). Females, on average, experienced less fatigue immediately following exercise. However, their fatigue values remained elevated throughout duration of recovery and thus was higher than males at 2.0 h.

### Cardiovascular measurements of recovery

**Fig. 5:**
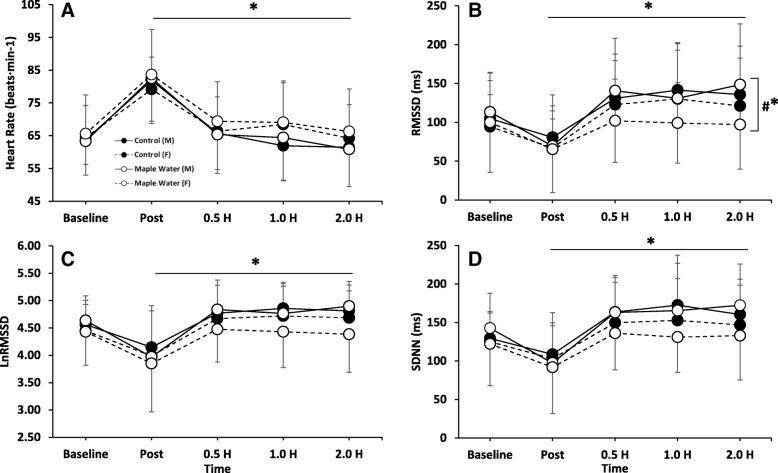
Measurements of Heart Rate and Heart Rate Variability at baseline, 2% Exercise-Induced Dehydration, and after ingestion of either Control or Maple Water in Males and Females (*n* = 26). **a** Heart Rate, **b** RMSSD, **c** Natural Log Transformed RMSSD (LnRMSSD), **d** SDNN. Data are mean ± SD.*p < 0.05 main effect for time, #* *p* < 0.05 interaction of sex by time

There were no significant (*p* > 0.05) condition by time interactions for HR, RMSSD, SDNN, or LnRMSSD (Fig. 5). There was also no significant (*p* > 0.05) effect of condition (MW vs. control) for HR, RMSSD, SDNN, or LnRMSSD. Significant time effects were observed for HR and HRV (Fig. 5). As expected, following exercise, HR increased with a corresponding decrease in RMSSD. For HR, both males and females returned to, or near to, baseline by the end of testing (Fig. 5a). However, only males returned to, or near to, baseline for RMSSD (Fig. 5b). Females had significantly (*p* < 0.05) lower RMSSD values at 0.5, 1.0, and 2.0 h, indicative of insufficient recovery (Fig. 5b). However, when RMSSD was natural log transformed (LnRMSSD; Fig. 5c), or when examining SDNN (Fig. 5d), while there was a trend for an effect of sex, no significant interactions or main effects of sex were found.

### Beverage analysis

Analysis of the maple water revealed a six-fold higher osmolality (81 ± 1.4 vs. 11 ± 0.7 mOsmol/kg, MW vs. control) and notable differences in electrolytes when compared to the control (Table 2). Analysis of the antioxidant potential (FRAP assay) indicated the maple water had an antioxidant potential nearly 4-fold higher than that of the control (3.9 ± 0.0 vs. 1.0 ± 0.1 mmol/L, *p* < 0.05).

**Table 2:** Nutritional content of Control (Maple Flavored Bottle Water) and Maple Water (MW)

Electrolyte	Control	Maple Water
Carbohydrates^a^ (g/L)	24	0
Calories^a^ (Kcal/L)	100	0
Calcium (mg/L)	1.90 ± 0.20	53.00 ± 5.00
Magnesium (mg/L)	0.25 ± 0.25	6.10 ± 0.80
Manganese^a^ (mg/L)	< 0.050	2.92
Potassium (mg/L)	0.23 ± 0.22	71.00 ± 6.00
Sodium (mg/L)	1.10 ± 0.90	< 0.100
Chloride (mg/L)	2.67 ± 0.07	9.00 ± 2.00
Carbonate (mg/L)	20.00 ± 10.00	45.00 ± 4.00
Phosphate (mg/L)	< 1.00	7.00 ± 2.00
Sulfate (mg/L)	1.70 ± 0.30	57.40 ± 0.40

Data are mean ± SD, ^a^from label

### Urine antioxidant analysis

**Fig. 6:**
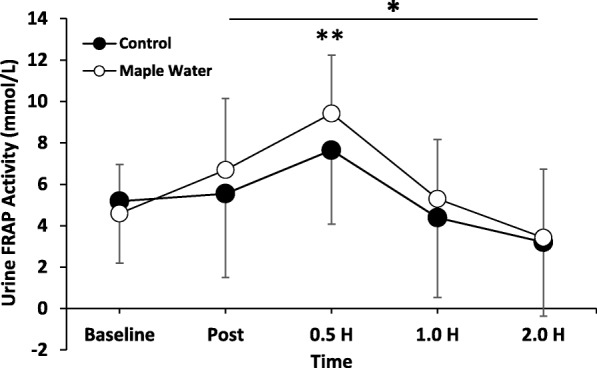
Measurements of Urinary Antioxidant Potential at baseline, after exercise at 2% Dehydration, and after ingestion of either Control or Maple Water (*n* = 14). Data are mean ± SD. *p < 0.05 main effect for time. **p < 0.05 control vs. maple water at time of peak

Analysis of urine for antioxidant potential revealed no significant interaction of condition (MW vs control) by time, nor a main effect of condition (Fig. 6, *p* > 0.05). However, there was a significant main effect for time, where urinary antioxidant capacity acutely increased post-exercise with a decay back towards baseline by 2.0 h. On average, following the consumption of Maple Water, FRAP analysis revealed greater antioxidant potential in the MW condition compared to the control, and when focusing on the peak response at 0.5 h, there was a significant difference between conditions (Fig. 6, *p* < 0.05).

## Discussion

The purpose of the present study was to investigate the efficacy of MW as a rehydration beverage following moderate intensity cycling in a hyperthermic environment. It was hypothesized that ingestion of 1 L of MW following 2% BW loss would enhance physiological and perceptual markers of hydration, reduce fatigue, and enhance recovery over that of maple-flavored bottled water. Despite a higher osmolality and electrolyte content, the current study revealed no significant differences between MW and control on rehydration. However, likely due to differences in osmolality, thirst sensation remained elevated following consumption of MW, indicative of a potential for enhanced rehydration under ad libitum conditions. When sex differences were explored, on average, females had lower urine volume and output, resulting in tendency for greater ΔBW, but more concentrated urine, as indicated by higher UOsm, which taken alone could be suggestive of impaired rehydration. However, the greater ΔBW and lack of sex differences in the SOsm suggests a potential sex specificity in the mechanisms of rehydration, but further controlled work on these potential sex differences is warranted. Additionally, we observed differences in the kinetics between urinary and salivary measurements; salivary peaks occurred immediately post-exercise while urinary peaks were delayed at 0.5 h post-rehydration. Lastly, MW demonstrated a higher antioxidant potential (AP), which translated into elevated AP measured in the urine. Collectively, MW is a natural rehydration beverage with electrolytes and antioxidant potential but failed to enhance rehydration over water alone. Further work is needed to elucidate the sex differences, and temporal variations between biological compartments, during rehydration.

### Rehydration: Carbohydrate-electrolyte sports beverages

Interventional studies on rehydration are relatively abundant in the literature [16–21], with the majority of these studies focusing on carbohydrate-electrolyte rich sports beverages, and the general consensus is that sports beverages are likely more effective in restoring sweat-induced hydromineral loss then water alone. However, the present study observed no differential effects of MW over water in terms of hydration (Fig. 2). Although MW boasts a rich electrolyte profile, the primary mineral-salts are Ca^++^, K^+^, carbonate, and sulfate, while Na^+^ is negligible (< 0.1 mg/L) (Table 2). However, as Na^+^ is a principle electrolyte lost in sweat; as compared to sodium-rich carbohydrate-electrolyte sports beverages (e.g. 457 mg/L of Na^+^) the relatively low Na + concentration in MW might explain, in part, the relative inadequacy in rehydration of MW and highlight that rehydration is likely sensitive to electrolyte composition. Though, previous work has suggested carbohydrate, protein, and/or caloric content might influence fluid uptake and rehydration [20, 58], perhaps due to relatively modest electrolytes, electrolyte composition, osmolality (~ 80 mOsm/kg), carbohydrates and caloric content (Table 2), we observed no such benefit of MW.

Nevertheless, arguments have been proposed against the efficacy of sports beverages. Shirreffs et al. investigated rehydration following intermittent exercise-induced dehydration (1.9% BW loss) in the heat [18]. They observed attenuated mineral-salt balance, but not hydration status (% BW lost), with carbohydrate-electrolyte sports beverage. The authors speculated that salt-mineral replenishment may be inadequate with carbohydrate-rich electrolyte beverages, which is just as, if not more, important as hydration [18]. Additionally, increased gastrointestinal (GI) discomfort following sports beverage consumption has also been observed [23, 28]. GI distress, likely due to individual variability in sensitivities to relatively high carbohydrate boluses post-exercise, as well as reduction of blood flow to the GI tract during exercise, which may blunt absorption, may explain the impaired mineral-salt balance observed in the previous studies [18]. Furthermore, anecdotally there is apprehension as to the potential for excess chronic consumption of liquid calories as it relates to weight, and concerns regarding the use of artificial sweeteners and dyes, warrants further investigation into safer and natural alternatives.

### Rehydration: Natural alternatives

Accordingly, there is a growing interest in natural, organic rehydration substitutes such as coconut water (CW) [27–29]. One study explored the potential impact of CW on rehydration after participants exercised at 65% of their VO_2max_ at 32 °C and 53% RH, an experimental intervention similar to that of the present study [27]. Rehydration was not significantly improved with CW. Similarly, when male volunteers exercised at 60% of their VO_2_max in the heat (31 °C, 51% RH) until 2.8% BW loss, %BW regained, rehydration index, blood volume, electrolyte profile, net fluid balance, and serum osmolality were not significantly different between CW and carbohydrate-electrolyte rich beverage [29], and agrees with other studies of CW [27–29]. Interestingly, in both studies, subjects were still hypohydrated by the end of testing, suggesting, perhaps that the study design could be optimized and/or CW might not be an effective rehydration choice.

The current study was the first to investigate the rehydration properties of MW. Using a similar experimental procedure, in accordance with previous studies investigating CW [27, 29], we found that consumption of MW was not different from water in rehydration (Fig. 2). Given an absolute volume (1 L), similar changes in ΔBW, UC (~ 2 units), USG (~.015), UOsm (~ 300 mOsm·kg^− 1^), and SOsm (~ 60 mOsm·kg^− 1^) were observed in both conditions, which returned to, or near to, baseline by the end of testing. However, despite intake of 1 L, MW sustained significantly greater perception of thirst over time (Fig. 4a), possibly due to the near six-fold greater osmolality concentration which might stimulate thirst and/or the modest amount of carbohydrates present in MW [59], but these differences in CHO content and osmolality were not great enough to elicit greater rehydration with MW. While not directly quantified in the present study, no participants reported any GI distress in response to the MW. Further, given the purported antioxidant properties of maple syrup [34], we sought to characterize the antioxidant capacity of MW, and revealed that MW had an antioxidant capacity ~ 4-fold higher than our control, and supports prior work which demonstrated, via the DPPH assay, that maple water has antioxidant capacity [30]. The antioxidant capacity of MW, as determined by the FRAP assay, is on par with prune juice or grape juice [60]. Although endogenous antioxidants (e.g. glutathione peroxidase) exist, exercise induces free radical outflow from muscle that may overwhelm our endogenous defenses [61]. Analysis of urine antioxidant capacity, indicated that the acute exercise positively impacted antioxidant capacity, and MW enhanced this response at 0.5 h after ingestion of MW (Fig. 6). Finally, in contrast to our hypothesis, MW had no impact on fatigue (Fig. 4b).

Collectively, MW may be an accessible, and safer natural electrolyte-containing alternative to CW and sports drinks, particularly when consumed ad libitum. However, more work is needed to explore the impact of MW on hydration, such as larger populations, greater levels of dehydration, altered volume of intake (e.g. 120% of BW lost), measurement of plasma osmolality, and/or ingestion behavior (ad libitum vs. prescribed).

### Rehydration: Potential sex effects

The current body of literature on the effects of sex on hydration is relatively limited. When matched for aerobic fitness and body fat percentage, Sawka et al. [35] found no changes in HR, rectal and skin temperature between males and females following treadmill exercise at 20 °C 40% RH, 35 °C 40% RH, and 35 °C 79% RH performed in both euhydrated and dehydrated states [35]. Though, Eijsvogels et al. demonstrated in a relatively aged, and heterogeneous population that sex differences do exist in dehydration, which appear to be, at least in part, to differences in fluid intake [36]. Moreover, focusing on rehydration specifically, work by Maughan et al. indicated that rehydration was not impacted by the menstrual cycle following exercise-induced dehydration to ~ 1.8%, a threshold similar to that of the present study [21]. However, following exercise-induced dehydration, females in the current study had a significantly higher UOsm, and a trend for elevated USG (digital and analog), which remained elevated throughout the duration of testing (Fig. 2), which, taken alone, is suggestive of less effective rehydration. However, in the females, urine volume and cumulative output were significantly lower (Fig. 3), and ∆BW tended to be higher (Fig. 2), indicative of increased water retention and likely rehydration in women, though measurement of plasma osmolality would help discern possible sex differences in fluid shifts. These findings would agree with prior literature, where women are more likely to maintain plasma osmolality or develop hyponatremia due to fluid retention, then men who are more likely to become hypernatremic [36].

Given the limited research on the effects of sex on rehydration specifically, mechanisms behind these differences are not well understood. A tenable explanation is the hormonal fluctuations associated with the menstrual cycle or use of oral contraceptive pills [62], neither of which were controlled for the in present study, potentially obscuring the sex differences that are presently reported, and is limitation of the current study. Though, Maughan et al. found no effect of menstrual cycle phase in rehydration after exercise-induced dehydration [21], including the relatively low circulating hormone menstrual phase. Mechanistically, the variation in the hypothalamic-pituitary-gonadal axis over the menstrual cycle; specifically, estrogen and/or progesterone, may alter arginine vasopressin or its effect on the kidney, which ultimately influences hydration status [37, 38], and might explain the reduced, and concentrated, urine output (i.e. elevated USG and UOsm) in the females. Indeed, analysis of a subset of males and females urine for renin and aldosterone, revealed trends for greater renin in women, compared to men, but no such trend was observed in aldosterone. Importantly, if women were excluded and only the male data was analyzed the relatively minimal findings related to the rehydrating efficacy of maple water were unchanged, and thus the data remain combined. To our knowledge, our study might be the first to report sex differences in rehydration, using a relative work rate, and controlling for baseline hydration status, and based on our findings, there may be sex specificity in the mechanisms of rehydration. However, a limitation to the current study is that we did not control for menstrual cycle phase or OCP use, thus future studies are critically needed to specifically evaluate potential mechanisms of sex differences and potential influence of the menstrual cycle phase or oral contraceptive pill use impacts on rehydration, after exercise-induced dehydration.

### Cardiovascular recovery from dehydration: Potential sex effects

The current study also investigated the potential impact of MW and/or sex on rehydration and measures of recovery using heart rate (HR) and HR variability (HRV). As expected, HR and HRV significantly increased and decreased, respectively following exercise-induced dehydration in the heat (Fig. 5) and returned to near baseline values for both conditions. These findings are somewhat in contrast to previous work by Carter et al., who demonstrated, using frequency domain measures, that HRV was impaired in recovery from dehydration and exercise in the heat [57].

When analyzed according to sex, RMSSD, a measure of parasympathetic contribution to HRV, significantly decreased following exercise (~ 120 pre vs. ~ 70 ms post) in both conditions, but returned to near baseline values for males only. A gap in the literature exists in regards to the potential role of sex on recovery of HRV, especially as it relates to heat- and exercise-induced dehydration. Therefore, it is not well understood the mechanisms behind the CV effects observed in present study. As mentioned before, menstrual cycle associated variation of sex (i.e. estrogen and progesterone) and non-sex hormones (arginine vasopressin) may likely explain these differences. Specifically, arginine vasopressin may increase sympathetic activity, which may attenuate recovery of HR and HRV [63], as observed in the present study. In support of this concept, in a subset of participants, urinary renin levels tended to be elevated in women, perhaps contributing to the impaired HRV. However, a limitation to the present findings is that menstrual cycle phase or oral contraceptive use were not controlled for, potentially obfuscating these reported sex differences. Mendonca et al. demonstrated sex differences in RR interval variability, but unlike the present study, they found that women, in response to a supramaximal Wingate test performed in normothermia, had enhanced cardiac parasympathetic input and a cardioprotective response (higher variability) [64]. Future studies on the effects of sex on HRV measures, especially as it pertains to exercise in the heat and dehydration, and the potential role of menstrual cycle phase or OCP use are still needed.

### Comparisons of hydration measures

To the best of our knowledge, no consensus exists about the optimal way to assess hydration status, thus a battery of hydration markers ought to be employed [42]. Nude BW is a simple, quick, and practical assessment of hydration status, but is not without error or assumption [45]. While blood borne assessments of hydration such as plasma osmolality (POsm), a relatively invasive measure, have been suggested to be a critical measure of static hydration and may not be sensitive to modest levels of dehydration [43, 44] due to highly controlled internal homeostatic mechanisms. On the other hand, urinary based measures, such as UOsm, are considered valid assessments sensitive to modest dehydration [44], but can be biased by fluid ingestion. SOsm has also been suggested to be a valid and effective method with similar sensitivity [9, 46], but also can be biased, short term by fluid ingestion.

Although not a primary research question in the current study, given the relatively comprehensive assessment of hydration we performed a preliminary examination of the methods. Incongruent with the urinary based measures of hydration suggesting adequate rehydration, the ΔBW suggested incomplete rehydration, due to diuresis, (Fig. 2), and agrees with prior work [65]. Interestingly, while acute fluid ingestion is known to alter SOsm [66], the acute effect wanes 30 min post ingestion, thus, in the absence of further ingestion SOsm remained low, similar to urine, but still discordant to BW. Differences in kinetics between fluid compartments were also apparent. Specifically, salivary measures peaked immediately post-exercise, whereas urinary values had some latency peaking at 0.5 h (Fig. 2). Further, through using both analog and digital refractometers we found that the analog refractometer tended to overestimate USG, but tracked the digital refractometer almost exactly over time. To our knowledge we may be the first to report these findings, and such knowledge may be instrumental in the design of future studies, and/or applications where it may be desirable to perform rapid physiological determinations of hydration in clinical, field, or research settings.

## Conclusion

In conclusion, the current study found that Maple Water was not superior in rehydration despite a modest electrolyte profile. Though sensations of thirst remained elevated with MW, indicative of a potential for enhanced ad libitum rehydration. Antioxidant potential was higher in maple water and increased urinary antioxidant capacity. Further, there is likely sex specificity in rehydration in the mechanisms of rehydration, where females appear to inhibit diuresis to better preserve fluid volume. Further research is needed on the rehydrating efficacy of MW and in understanding the potential effects of sex, menstrual cycle phase, and oral contraceptive use on physiological parameters of hydration status in response to dehydration and rehydration.

## Abbreviations

AP: Antioxidant potential
BW: Body weight
CV: Cardiovascular
CW: Coconut water
h: Hour
HR: Heart rate
HRV: Heart rate variability
LnRMSSD: Natural log transformed RMSSD
MW: Maple water
RMSSD: Root mean square of successive differences
SDNN: Standard deviation of N-N intervals
SOsm: Saliva osmolality
UC: Urine color
UOsm: Urine osmolality
USG: Urine specific gravity
UV: Urine volume
